# Growth of crystalline WO_3_-ZnSe nanocomposites: an approach to optical, electrochemical, and catalytic properties

**DOI:** 10.1038/s41598-022-07951-5

**Published:** 2022-03-10

**Authors:** Insaaf Assadullah, Javied Hamid Malik, Adil Shafi, Radha Tomar

**Affiliations:** 1 School of Studies in Chemistry, Jiwaji University, Gwalior, 474011 India; 2 Environmental Research Laboratory, Department of Chemistry, Aligarh Muslim University, Aligarh, Uttar Pradesh 202002 India

**Keywords:** Chemistry, Materials science, Nanoscience and technology

## Abstract

In this study, novel growth of WO_3_-ZnSe nanocomposites was carried out by a simple, low-cost hydrothermal process under subcritical conditions and is reported for the first time in just 5 h. The products were characterized in detail by multiform techniques: X-ray diffraction, scanning electron microscopy (SEM), optical studies, and Fourier transform analysis. The influence of ZnSe on the structural, morphological, compositional, optical, and catalytic properties of WO_3_ is demonstrated. The WO_3_ metal oxide material is grown in a hexagonal crystal structure with wide-band-gap and has been modified by ZnSe to form a composite nanostructures in the nanoscale range. The electrochemical properties of the prepared materials were studied by cyclic voltammetry, which revealed that the synthesized material exhibited remarkable electrochemical supercapacitive activity. Moreover, the composite nanostructures showed excellent photocatalytic activity for degradation of phenol and almost 93% of phenol was degraded with good recyclability and stability. According to The International Commission on Illumination (CIE), the synthesized nanomaterial shows blue emission and is suitable for blue LEDs.

## Introduction

Environmental contamination and energy crisis are major issues that we are confronting in every facet of biosphere. Rapid industrialization and expanded urbanization has resulted in disturbing natural harmony by adding harmful pollutants into the environmental mediums. Various pollutants such as drug effluents, phenols, pesticides, dyes, fungicides, and phthalates have been added to soils and water bodies and have deteriorated their quality and standard. In addition to pollutants, the prevalence of different endocrine disruptors, such as 17-β-estradiol and bisphenol A (BPA) in environment has resulted in serious health problems. Researchers across the globe are devoting their efforts to fabricate modern and advanced techniques to decontaminate and mitigate the harmful effects of these pollutants. Among the advanced techniques, photocatalytic degradation using semiconductor nanoparticles is considered as sustainable technique to degrade so many organic and inorganic pollutants. Although, photocatalysis is considered as signature technology for degrading pollutants but the application is limited by wide band gap semiconductors photocatalysts.

Semiconductor nanoparticles are receiving recognizable attention in photocatalysis, because of their awesome morphology and peculiar properties. The peculiar features of semiconductor photocatalysts are remarkable assessed for reclamation and remediation of contaminated environment. However, these nanoparticles suffer serious drawback of light absorption in ultra violet region^1–9^.

Moreover, the nano-based materials have shown excellent applications in energy generation and energy storage due to large specific surface area, great chemical stability, superb mechanical properties, and excellent electron mobility. Various transition metal oxides have been utilized for energy storage and have used as electrode materials in supercapacitors. But the problem with the transition metal oxide is limited DC conductivity and aggregative behavior^10–12^.

In recent years, tungsten oxide (WO_3_) has attracted the interest of many researchers because of its versatile nature and multiple properties. WO_3_ is an n-type semiconductor and potential visible light photocatalyst. It is regarded as one of the most promising semiconductor photocatalysts for degrading organic compounds due to its small bandgap (which varies from 2.4 to 2.8 eV), inexpensive cost and high stability in aqueous solutions under acidic conditions^13^. WO_3_ has been used as a potential material in electrochemical, photocatalytic, and gas detecting gadgets^9^. Moreover, a new report has shown that tungsten oxide has noteworthy biophotocatalytic properties alongside its nonperilous nature that has shown its potential applications in nanobioinnovation^14–18^. Zinc Selenide (ZnSe) was developed as the most encouraging photocatalytic material with a band gap of 2.7 eV at room temperature and enormous excitation restricting energy of 21 meV. Recently, the nanostructures of ZnSe have received much consideration because of their outstanding redox properties. Ongoing investigations showed that these ZnSe nanostructures display great photocatalytic activities and retain the edge of apparent light.

To improve the activity of these individual nanostructures, the development of ZnSe-WO_3_ nanocomposite was performed, which not only improves the absorption of visible light but also efficiently isolates photogenerated charges. The synthesis of heterojunctions with suitable arrangement of conduction bands and valence bands prompt diminished recombination of charge carriers^19–22^. Thus, overlapping band edges at the interface promotes charge transfer and higher light absorption. Numerous biomolecule-supported materials, such as chitosan-GO^23^, and chitosan-TiO_2_^24^ and heterojunctions, such as ZnO–TiO_2_, TiO_2_–Cu2S^25^, and ZnO–ZnSe^26^, are being utilized to remove environmental pollutants through adsorption and photocatalysis.

In the present study, we report the cost-effective synthesis of WO_3_-ZnSe nanocomposites and have applied them for multiple applications, such as electrochemical, photoluminescent, and photocatalytic applications. The fabrication of composite material has an advantage over individual counterparts in light absorption, redox activity and interfacial charge transfer. The photocatalytic activity was checked with degradation of phenol and in electrochemical activity, supercapacitive behavior was checked. The WO_3_-ZnSe composites showed excellent photocatalytic and good capacitive behavior with 93% of phenol degradation in 105 min.

### Fabrication of the WO3-ZnSe nanostructures

All the substances were analytic-grade reagents without further distillation.WO_3_ nanoparticles were synthesized under hydrothermal conditions. The experimental details were as follows: 2 g sodium tungstate dihydrate (Na_2_WO_3_·2H_2_O) was dissolved in 40 mL of distilled water. HCl solution was added dropwise until the color changed from transparent to light yellow with constant stirring for 30 min. This colored liquid was then transferred into a Teflon-coated autoclave and put in a hot air oven for 4 h at 180 °C. Then cool the former at room temperature. The sample was then filtered and washed several times with ethanol and distilled water. The collected sample was dried in an oven at 50 °C.

ZnSe nanostructures were synthesized via a hydrothermal approach as reported earlier^27^. The WO3-ZnSe nanocomposite was prepared by the hydrothermal method. The preparation was performed by mixing WO_3_ nanoparticles into the ZnSe solution. The whole sample was transferred into a Teflon-coated autoclave in a hot air oven at 180 °C for just 5 h. Cool the autoclave naturally and filter the particles. The collected sample was washed several times with condensed water and ethanol. Dry the particles for the characterization part.

### Characterization techniques

The Rigaku Miniflex 600 diffractometer was used to perform the diffraction patterns of the synthesized material. In order to see the particle size, morphology and elemental composition analysis scanning electron microscopy (SEM), and energy-dispersive spectroscopy (EDS) was employed, by a field emission-scanning electron microscope (FESEM) (Philips Model-Quanta 200 FEG). For optical studies an UV–Visible spectrophotometer (UV2450 Shimadzu) was used. For photoluminescence, a spectrofluorophotometer (RF 6000, Shimadzu) was employed.

## Results and discussion

**Figure 1:**
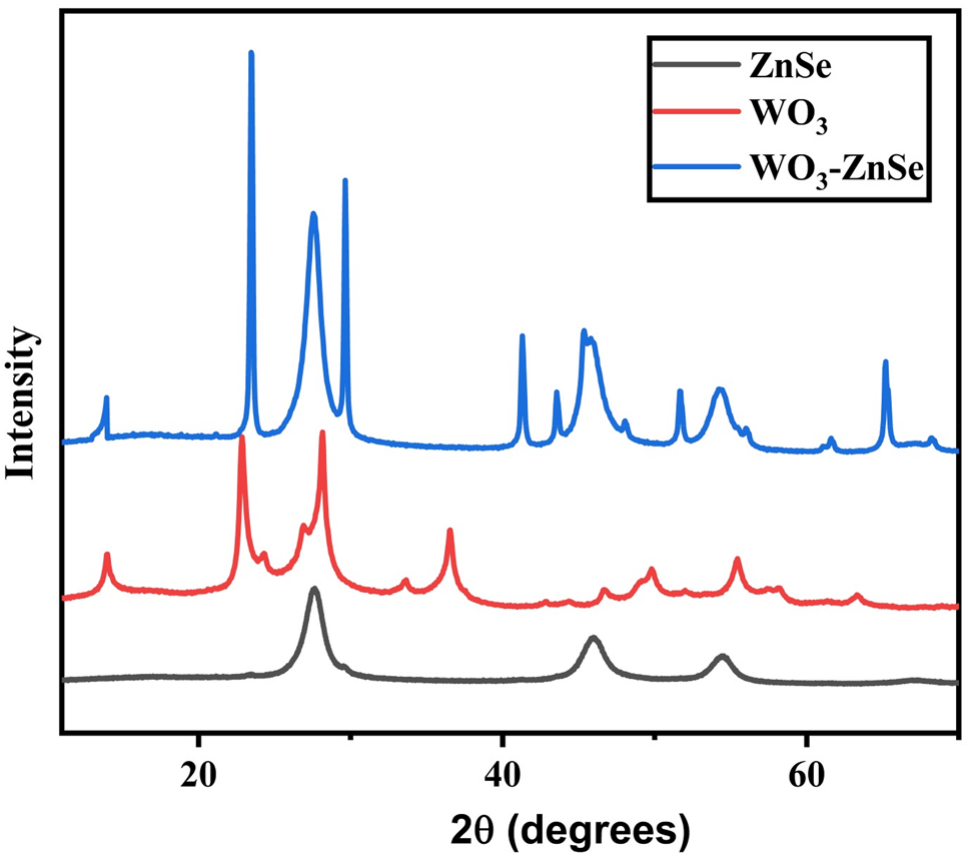
Shows the XRD patterns of the ZnSe, WO_3_, and WO_3_-ZnSe composites.

The crystal structure of the composite material was investigated by X-ray diffraction. Figure 1 shows the XRD patterns of the ZnSe, WO_3_, and WO_3_-ZnSe nanocomposites. The peaks for ZnSe are indexed and perfectly match with the cubic phase. The lattice parameters were calculated to be a = 5.5 Ǻ and matched well with the reported values for cubic ZnSe^27^. The XRD pattern of WO_3_ achieved by hydrothermal rote at 180°Cfor 5 h, has major peaks at 13.79, 22.78, 24.37, 26.84, 28.21, 33.61, 36.57, 42.89, 44.44, 46.54, 49.11,55.32, 58.35, 63.47, 70.65, 77.70, 80.31 degrees and consequently the corresponding planes are (100), (001), (110), (101), (200), (111), (201), (300), (211), (002), (301), (202), (400), (401), (222), (402), (420) which ensure the formation of hexagonal phase of WO_3_ nanoparticles and they match well with the (JCPDS card no. 080634). The maximum of the major peaks are sharp, which represents the high crystallinity of WO_3_ nanoparticles. No, hydrated WO_3_ peaks were found in the sample, indicating the high purity of WO_3_. The average grain size of the synthesized nanostructures was found to be between 3 and 30 nm, shown in Table 1 and was calculated by using the Scherer formula.

**Table 1:** Size of synthesized nanoparticles.

Sample	2θ	d(hkl)	β	D
ZnSe	28	3.22	0.547	15.64
WO_3_	13.79	6.41	0.77	10.86
WO_3_-ZnSe	13.8	3.281	0.671	12.45

$$D=0.9\times \frac{\uplambda }{\mathrm{\beta cos\theta }}.$$

Here, D is the average size of the nanoparticles, n is the dimensionless shape factor (0.9), λ is the wavelength of incident X-ray (λ = 1.54 Å), β is the full width at half maximum (FWHM) of the diffraction peak and θ is the angle of diffraction.

The XRD pattern of the WO_3_-ZnSe nanocomposite, confirms the presence of both WO_3_ and ZnSe. No, important transformations are seen in the XRD patterns of the pure WO_3_ and WO_3_-ZnSe nanocomposites. This means that the phase purity of WO_3_ is maintained after composite formation with ZnSe, which is preferred for photocatalytic activity.

### FTIR analysis

**Figure 2:**
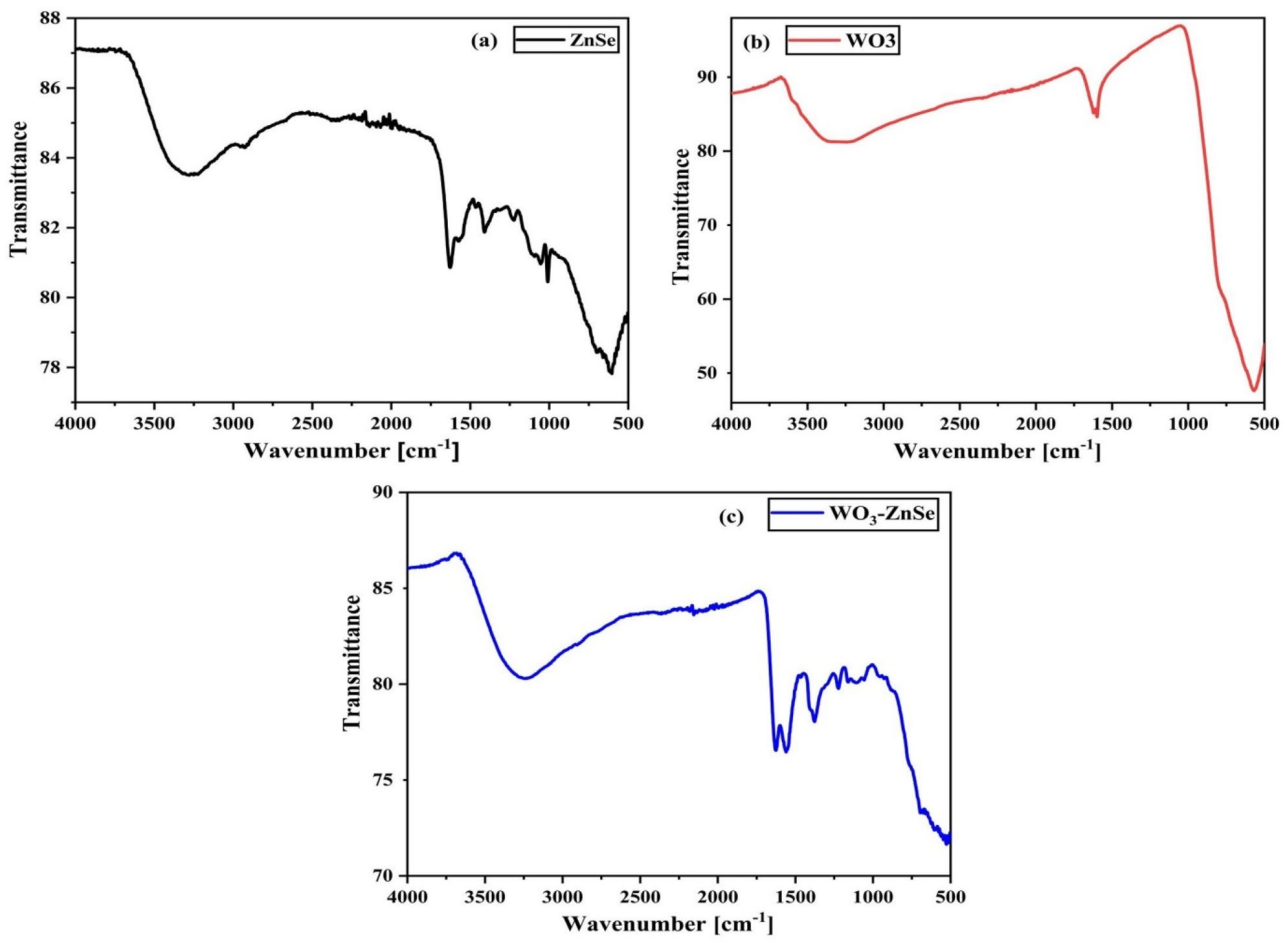
FTIR spectra of (**a**) ZnSe, (**b**) WO_3_, and (**c**) WO_3_-ZnSe.

Figure 2 displays the FTIR spectra of ZnSe, WO_3_, and WO_3_-ZnSe (a, b, c) nanocomposites respectively were recorded in the region 500–4000 cm^−1^. The broad absorption peaks in (a) between 550 and 650 cm^−1^ belongs to ZnSe vibrations which resemble with the previous reported ZnSe nanoparticles. The crests at 760 and 793 cm^−1^ in (b) are assigned to O–W–O bond stretching vibration modes and the crests at 3446 cm^−1^ correspond to the (O–H) stretching mode owed by the adsorption of the H_2_O molecules^28–31^. The crests at a 3417 cm^−1^ match O–H stretching. The FTIR analysis confirms the formation of WO_3_-ZnS.

### SEM and TEM analysis

**Figure 3:**
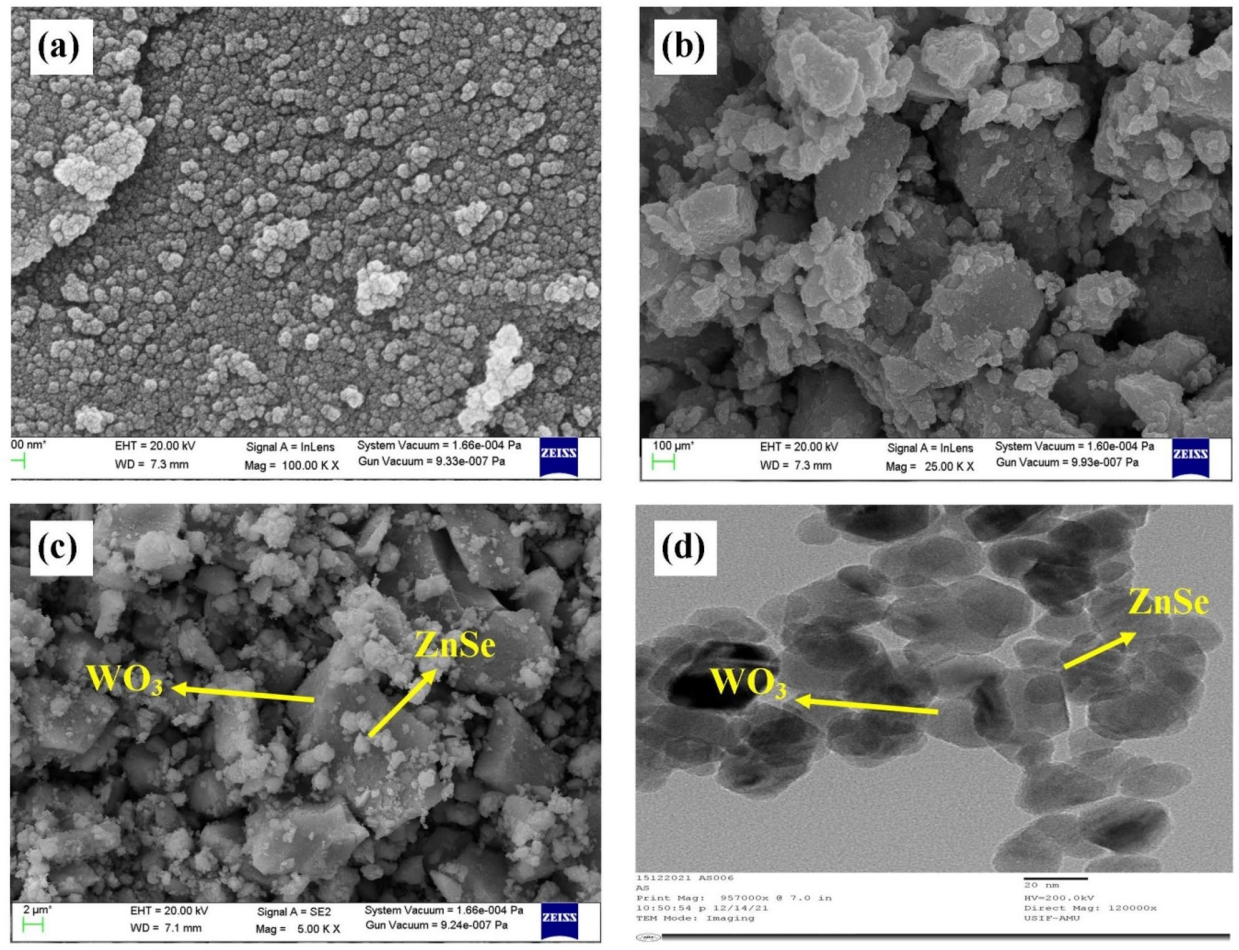
SEM micrographs of (**a**) ZnSe, (**b**) WO_3_ and (**c**) WO_3_-ZnSe, (**d**) TEM image of WO_3_ ZnSe nanostructures.

The morphology and particle size of the sample were studied by scanning electron microscopy. Figure 3 represents the SEM micrographs of ZnSe (a), WO_3_ (b) and WO_3_-Znse (c) nanoparticles. WO_3_ nanoparticles have an irregular structure or solid rock-like structure while ZnSe particles are spherical. It is clear from the SEM images that the spherical nanostructures are dispersed on the surface of WO_3_ nanoparticles, hence increasing the surface area of the synthesized material, which enhances the catalytic properties of the material. The outer state and morphology of the sample can play a major role in improving photocatalytic activity. When the electrons and holes are surrounded in surface states, the nonlinear overlaps of charge carriers are reduced, and their recombination is further stunted due to the localized nature of the surface area. In the present study, the formation of associative rock-like WO_3_ and spherical ZnSe particles will lead to the formation of efficient photocatalyst for the interfacial charge transfer and separation of photoexcited electron–hole pairs. Thus, the synthesized material can be favorable for photocatalytic activity.

The further insight into the surface morphology and structure was ascertained by Transmission electron microscopy (TEM). It is clear from Fig. 3d that spherical particles of ZnSe are irregularly dispersed on the surface of WO_3_ nanoparticles. The shapes of particles are clearly seen to be spherical anchored on rock like nanostructures having dimensions in the nanometer scale. The irregular dispersion of crystalline ZnSe particles on the surface of WO_3_ nanostructures may increase the surface active sites and enhance catalytic activity.

**Figure 4:**
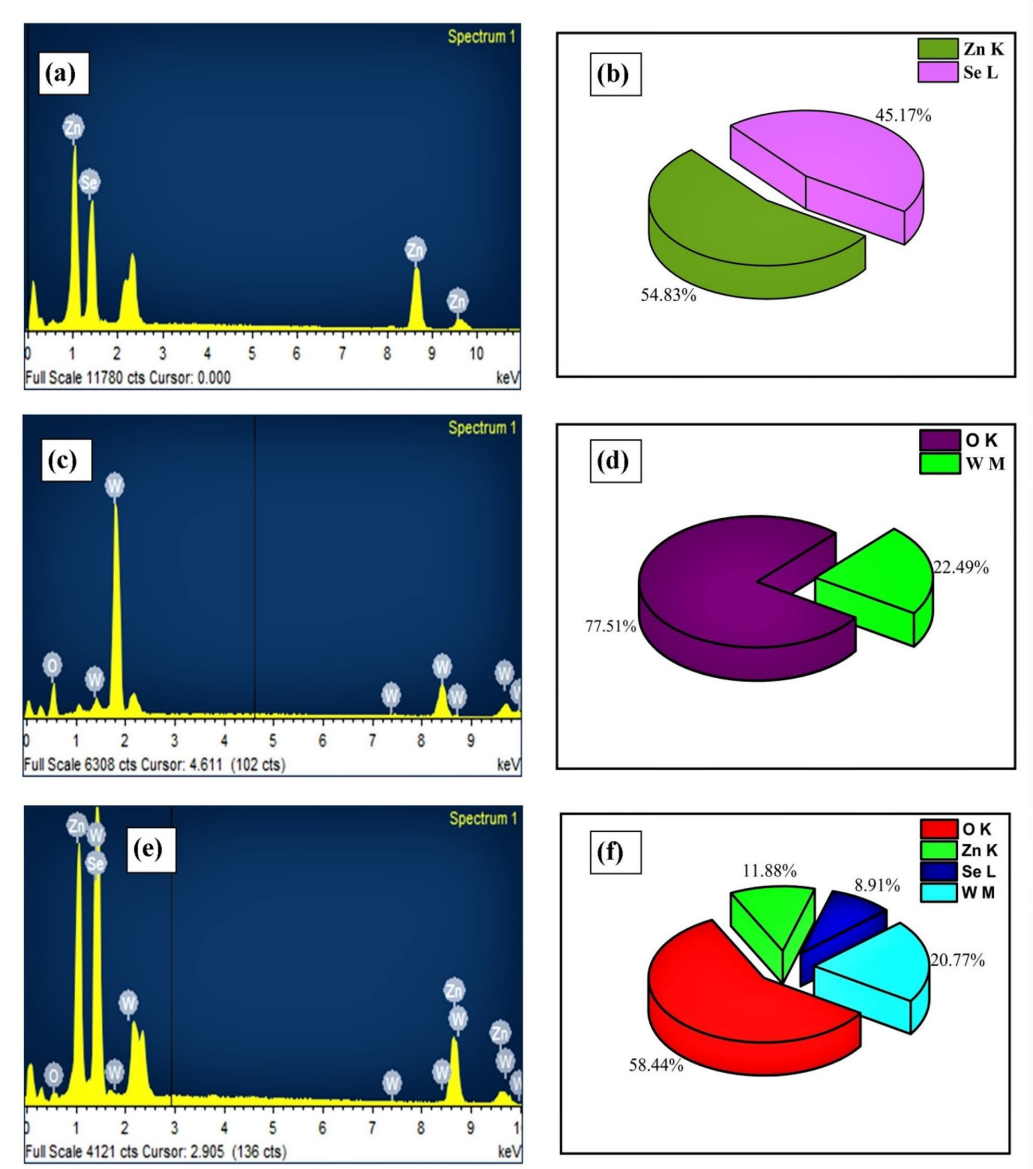
Shows EDS and elemental composition of ZnSe (**a,b**), WO_3_ (**c,d**), and WO_3_-ZnSe (**e,f**).

To determine the elemental composition of the fabricated samples, energy dispersive spectrometry (EDS) was employed. The EDS arrangements and elemental composition of ZnSe, WO_3_ and WO_3_-ZnSe nanoparticles are shown in Fig. 4a–f respectively. The main elements in the samples were Zn, Se, O and W. No, and other elements were detected, thus, displaying the high transparency of the sample.

### Optical analysis

**Figure 5:**
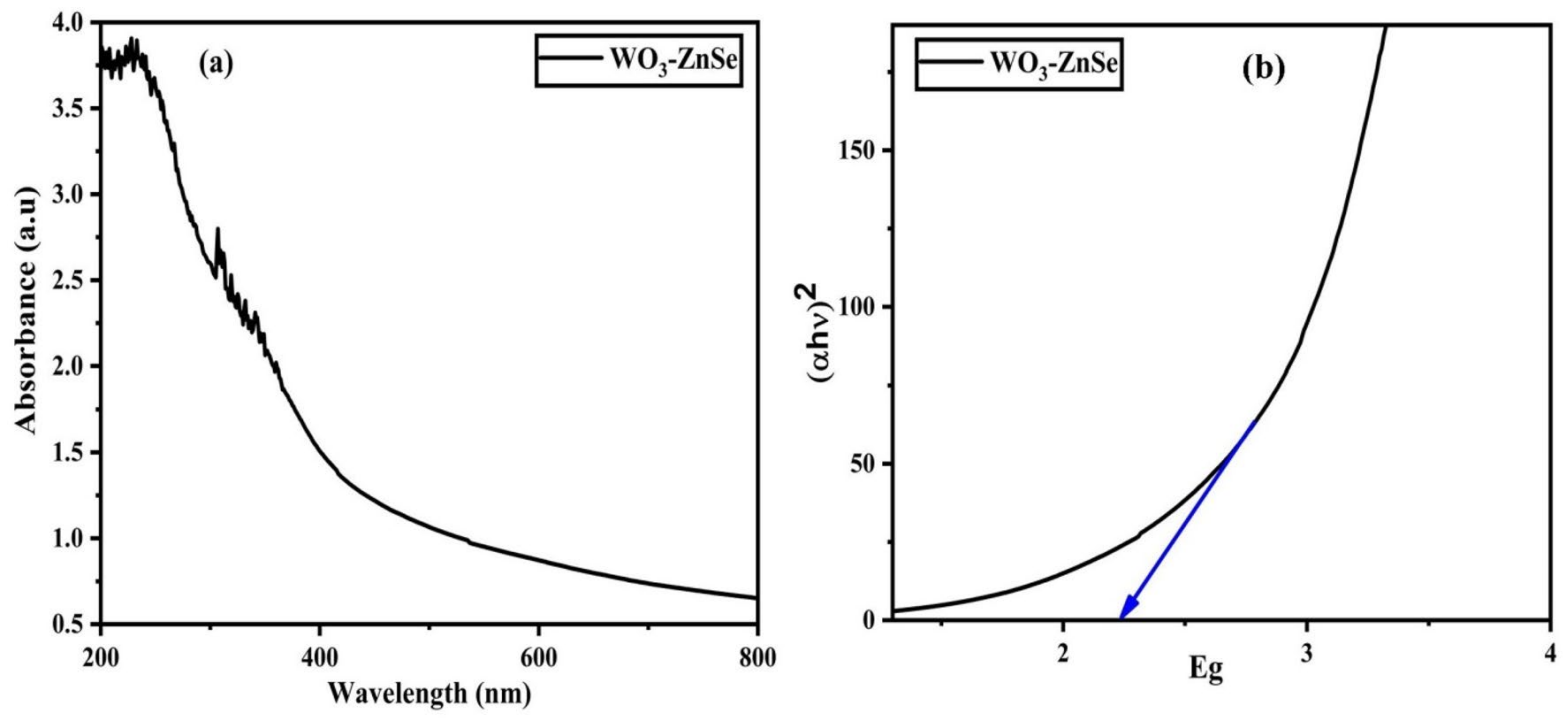
Absorbance spectra (**a**) and Tauc plot (**b**) of WO_3_-ZnSe nanostructures.

With the help of a UV–Visible spectrophotometer and spectrofluorometer, the light absorption spectra of the ZnSe and WO_3_ nanostructures revealed an absorption edge at 650 and 400 nm and the corresponding estimated band energy gap values are around 2.5 and 2.7 eV, respectively^27,32^. This wavelength is attributed to the band edge value of the material. The optical properties of the as-synthesized nanoparticles were assessed by UV–Vis absorption and matched well with their indirect bandgap energy. On the other hand there is a sharp increase in the absorbance for WO_3_-ZnSe nanostructures at 450 nm displayed in Fig. 5a. The Tauc plot of the bandgap for WO_3_-ZnSe nanostructures is displayed in Fig. 5b and it is clear from the figure that there is slight decrease in the value of bandgap due to increase in in particle size of the composite nanostructures. The Optical band gap values can be calculated by using the wood and tauc equation.

$${(\mathrm{\alpha h\nu })}^{2}=\mathrm{A}\left(\mathrm{h\nu }-\mathrm{Eg}\right).$$

Here, (α) is the absorbance, (h) is the Plank’s constant, (v) is the corresponding frequency, (Eg) is the optical bandgap, and the value of ‘n’ depends upon the transition of electrons.

### Electrochemical analysis

**Figure 6:**
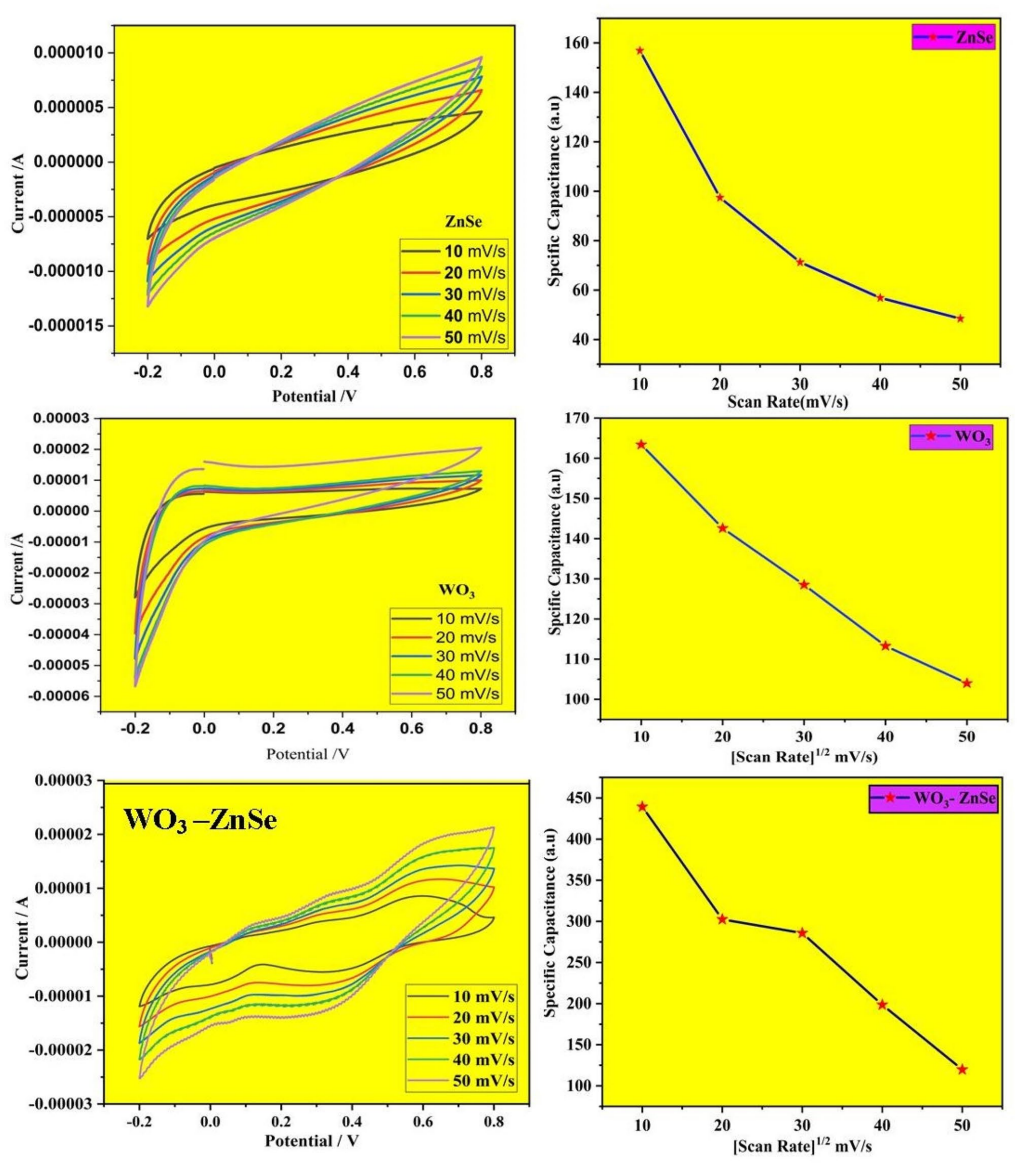
Electrochemical properties of ZnSe, WO_3_, and WO_3_-ZnSe nanocomposites.

Electrochemical analysis, such as CV and specific capacitance measurement, were used to study the charge-storage performances of ZnSe, WO_3_ and WO_3_-ZnSe nanocomposites in three-electrode setups displayed in Fig. 6. The fabricated samples were homogeneously dropped cast on the working electrode (made up of glassy carbon) at room temperature. Ag/AgCl and platinum wires were used as reference and counter electrodes, respectively. All three electrodes were dipped in 0.5 M H_2_SO_4_ solution, which is used as an electrolyte in an electrochemical cell. Figure 6 display typical CV plots from 10 to 50 mVs^−1^. The pair of redox reactions that are unseen in electrical double-layer capacitors is obvious in the CV curve and corroborate its battery type behavior, which is the significant requirement of the pseudocapacitive mechanism^11,33,34^. The studies also showed that the redox reaction is a diffusion-controlled progression, which may be the main cause of the pseudocapacitive behavior with increasing scan rates. Cyclic voltammetric curves are affected by diffusion-controlled and kinetic–controlled processes. If a redox process is only affected by diffusion, the peak potential should generally be independent of the scan rate. If the electrode kinetics is dominant, the peak potential is affected by voltammetric changes due to increasing scan rates. The shape of CV curves change little and the area enclosed increases with the increasing of scan rate, this demonstrates that the WO_3_-ZnSe nanocomposites have good electrical conductivity and reversibility. WO_3_ nanoparticles showed a less capacitance but after making composites with ZnSe, the relative specific capacitance increased by a large amount which are clearly shown in the Fig. 6.

### Electrochemical impendence spectroscopy analysis

**Figure 7:**
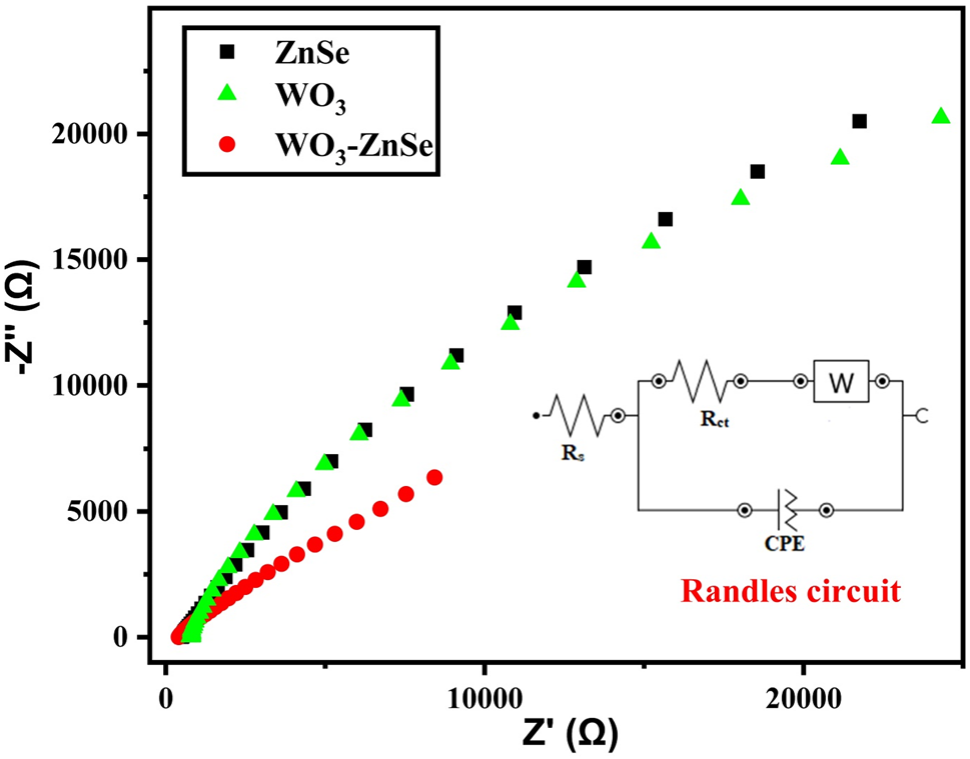
EIS analysis of ZnSe, WO_3_, and WO_3_-ZnSe nanocomposites.

Electrochemical impedance spectroscopy (EIS) was done to study the rate of interfacial charge transfer and dynamic kinetics of electrode reaction. The Nyquist plots for ZnSe, WO_3_, and WO_3_-ZnSe nanocomposites are shown in Fig. 7. Impedance is the power that contradicts electrical flow, and it is estimated in units of resistance (Ω). The genuine part (Z) versus the fantasy part (Z) of the impedance, which selects the interphase resistance between the functioning cathode and the electrolyte, was employed to plot EIS spectra. The R_ct_ of composite WO_3_-ZnSe was found to be lower than pure oxide and selenide counterparts. The lower resistance of composite material is the clear indication of better charge transfer across the interfaces and hence increased activity. Among the pure counterparts WO_3_ has seen little charge drag was seen more conductive than ZnSe. The linear EIS plots of the materials dictate the capacitive behaviour of the electrochemical system. The kinetic parameters have been deduced by fitting data in Randle’s Circuit (Inset Fig. 7). The values of simulated kinetic parameters have been presented in Table 2. It is clear from Table 2, that composite nanostructure has less Rct then ZnSe and WO_3_. The lower resistance and good CPE value dictates excellent electroactivity and good capacitive behaviour of composite WO_3_-ZnSe nanocomposites.

**Table 2:** Simulated kinetic parameters obtained from linear fit.

Material	R_ct_ (kΩ cm^2^)	R_s_ (Ω cm^2^)	CPE (μMho)
ZnSe	24	62.3	6.0
WO_3_	19	62.3	12
WO_3_-ZnSe	12	62.3	18

### Electrochemical active surface area (ECSA)

**Figure 8:**
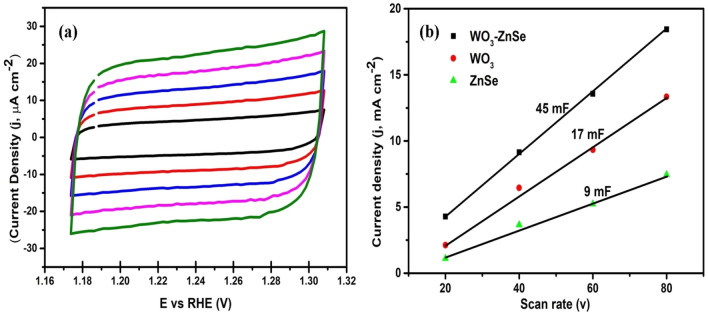
(**a**) Cyclic voltammogram’s at different scan rates in non-faradic region. (**b**) Plot of Capacitive current vs. scan rate.

The active surface area of the nanostructures was determined by electrochemical double layer capacitance (EDLC, C_dl_) (Fig. 8a,b). The active surface area was determined by executing cyclic voltammetry in non-faradic region. The current density (J) was plotted with scan rate (v) and from the slope of the plot double layer capacitance (C_dl_) was deduced. The slope is considered to be equal to double layer capacitance which in turn is considered to be proportional to electrochemically active surface area. The Cyclic voltammogram’s are depicted in Fig. 8a which advocates good capacitive behaviour of prepared nanostructures. From the linear plot Fig. 8b of current density (J) vs scan rate (v), the ECSA for WO_3_-ZnSe, WO_3_ and ZnSe was calculated to be 45 mF cm^−2^, 17 mF cm^−2^ and 9 mF cm^−2^. It can be seen from the values that composite nanostructures has got increased surface area followed by WO_3_ and ZnSe. The increased surface can lead to more active sites and enhanced photocatalytic activity. Moreover, the capacitive behaviour is also dependent on surface area and will increase upon surface increment.

### Photoluminescence

**Figure 9:**
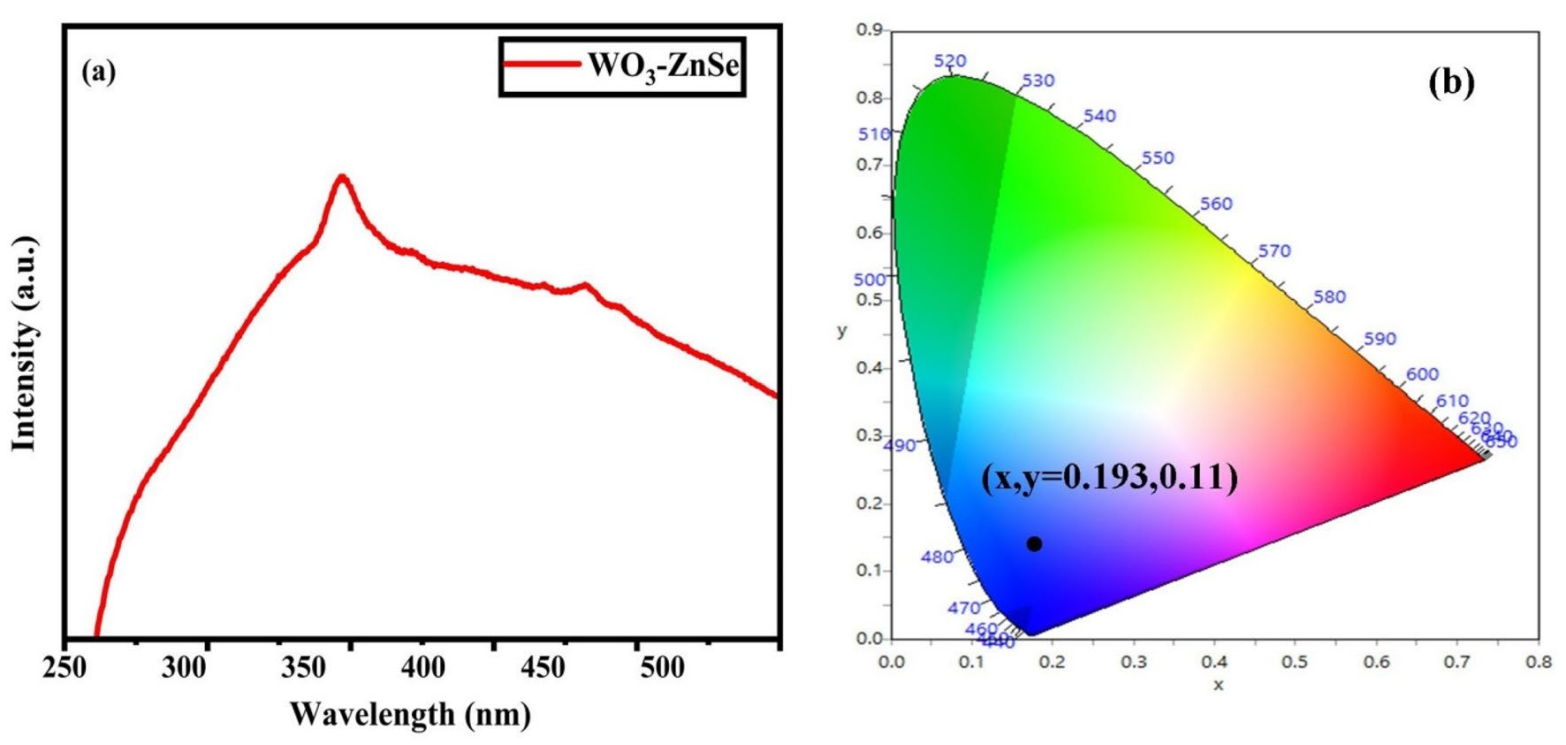
(**a**) PL spectra and (**b**) CIE plot of WO_3_-ZnSe composites.

To spot the photoluminescence in the WO_3_-ZnSe nanocomposites, the sample was subjected to an RF spectrofluorometer. Figure 9a represents the PL spectrum and comprises two peaks. The WO_3_-ZnSe nanostructures display a low intensity PL signal due to slower recombination of electron–hole pairs in the range of 320 to 400 nm, which is responsible for enhancing the catalytic properties of the nanocomposites. The two emission peaks at 364 and 381 nm were attributed to electron–hole radiative recombination. Figure 9a shows the PL spectra of the WO_3_-ZnSe nanoparticles with an excitation wavelength of 240 nm. The WO_3_-ZnSe nanoparticles can expose a wide PL band in the range of 320 to 400 nm. Two PL emission peaks at 364 and 381 nm were observed. The peak at 364 nm signifies near band edge emission (NBE). To further consider the luminescence properties of WO_3_-ZnSe nanocomposites, the color coordinates of the sample according to emission spectrum were calculated by CIE diagram which is shown in Fig. 9b. The International Commission on Illumination (CIE) coordinates are located at (x, y = 0.193, 0.11) and it is observed that this point lies in the blue region, thus showing that the material can be used in clinical applications and blue LEDs. This improvement managed to the power-efficient displays for iPads, computers, TVs, cell phones, etc., and many other electronic wonders of the current world.

### Photocatalytic activity

**Figure 10:**
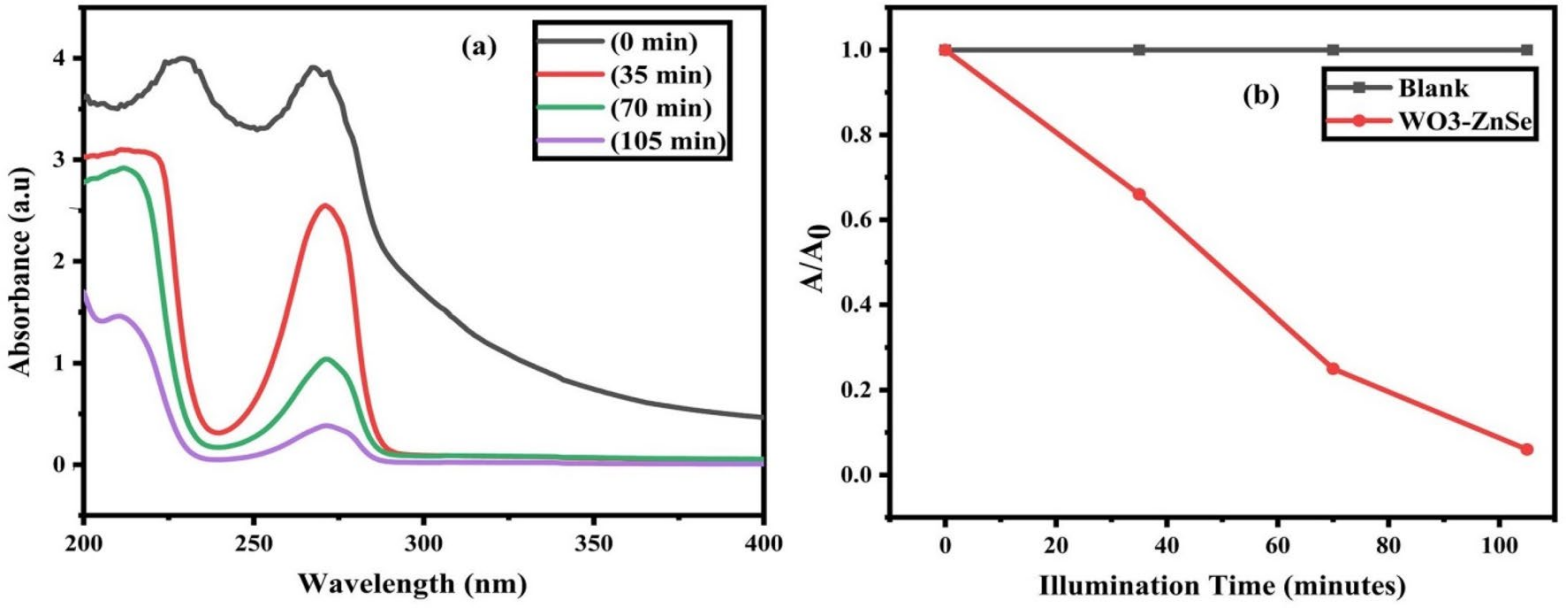
(**a**) Time-dependent absorbance spectra and (**b**) concentration curves of blank (without catalyst) and synthesized samples.

The photocatalytic activity of the synthesized WO_3_-ZnSe nanostructures was calculated based on the photocatalytic degradation of phenol solution under UV–Visible light irradiation, as shown in Fig. 10. The photodegradation of the carbon-based compound was detected by the absorption peak at 271 nm. The compound was almost fully degraded within 105 min and the degradation percentage reached almost 93%. These results suggested that the WO_3_-ZnSe nanomaterial can act as an excellent photocatalyst for the degradation of organic pollutants. The nanostructures were explored for photocatalysis by investigating the degradation of an organic compound phenol under UV–Visible light illumination. The time-dependent absorbance spectra of the phenol solution treated with catalyst are shown in Fig. 10a solution as per the Lambert–Beer law. The decrease in concentration was calculated using the Lambert–Beer proportionality law as:

$$\frac{Ct}{C0}=\frac{At}{A0}.$$

The starting and final concentrations are C_0_ and C_t_, respectively. This proportion was plotted against the illumination time shown in Fig. 10b for the phenol solution treated with the WO_3_-ZnSe catalysts. From the concentration plots, it is obvious that the decrease in concentration is very rapid as soon as the catalysts are introduced into the solution in comparison to the blank solution. From the spectra Fig. 10a, it is visible that the phenol bands showed a gradual decrease without any shift. From the observations, it is determined that the concentration drop is due to the decolorization process. The declaration percentage was calculated using the following relation:

$$\mathrm{Decolaration\%}=\left(1-\frac{\mathrm{At}}{\mathrm{A}0}\right)\times 100.$$

After, 105 min of light illumination, the nanostructures of WO_3_-ZnSe decolored approximately 93.3% of the phenol. In two steps, the possible mechanism for photodecolarization can be explained. Highly reactive species (free radicals) are produced at first, and the next step is to have these free radicals oxidize the adsorbed phenol molecules (see Fig. 10). The catalyst is bordered by the OH molecules and reduces them to negatively charge when they are dispersed in the phenol solution. Due to electrostatic interactions, phenol is readily adsorbed across catalyst surfaces since it is weakly acidic. When exposed to a light source, photons with energy equal to or greater than the bandgap of the semiconductor are produced. Electron–hole pairs are formed as a result of the absorbed photons. Electrons are drawn to the conduction bands, while holes are drawn to the valence bands. The holes are consumed by absorbed hydroxyl groups, which are then converted into extremely active hydroxyl free radicals. Conduction electrons transform molecular oxygen to generate superoxides, which then react with water molecules to form peroxides.

### Stability of the photocatalyst

**Figure 11:**
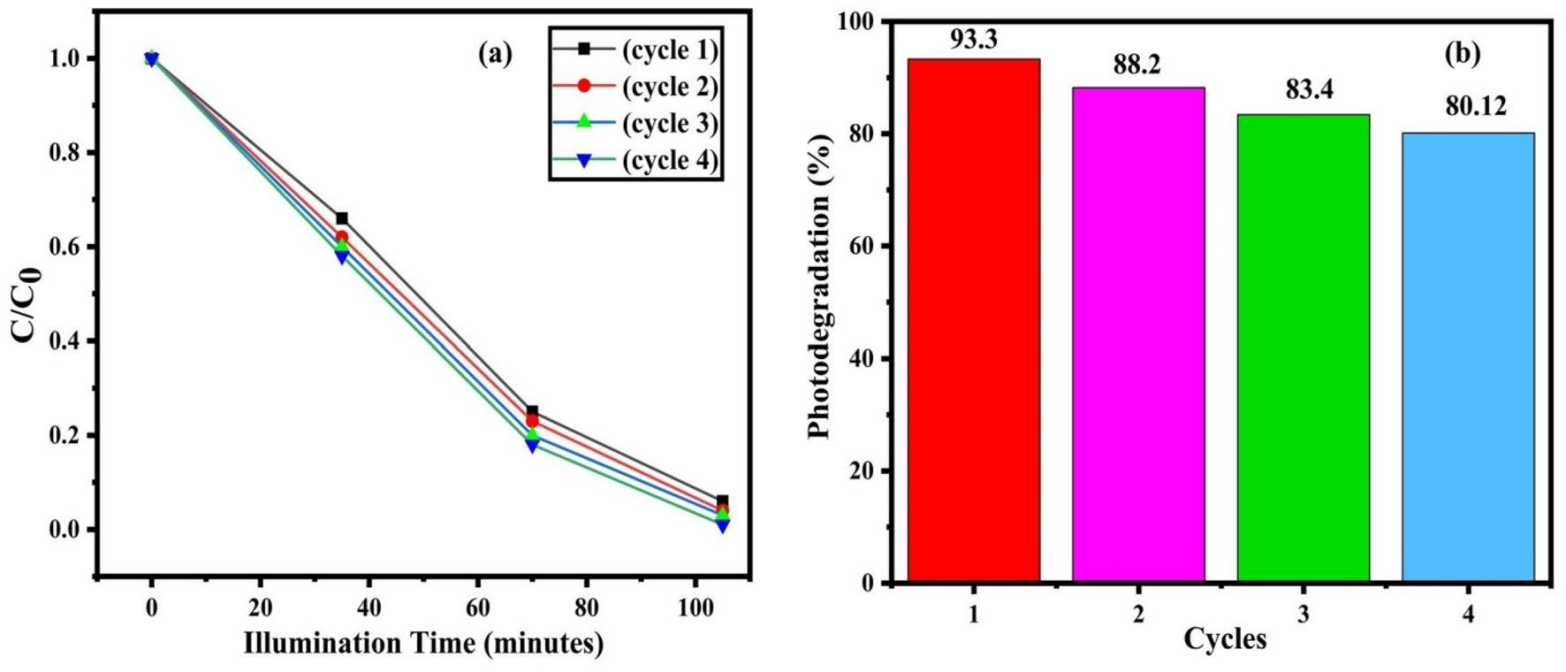
(**a**) Time-dependent concentration curves and (**b**) degradation percentages for four successive degradation cycles.

The reuse test was utilized to measure the stability of the synthesized photocatalyst, and the outcomes are introduced in Fig. 11a,b for WO_3_-ZnSe nanocomposites. Even after the fourth cycle, the photocatalyst remained remarkably steady. The photocatalytic activity of both materials was reduced in the third cycle and thereafter remained relatively stable, according to the findings. The lower proportion of degradation may be related to successive degradation cycles.

## Conclusion

Novel growth of WO_3_-ZnSe nanocomposites was carried out by a simple, low-cost hydrothermal process under subcritical conditions and is reported for the first time. The comprehensive morphological characterizations revealed the crystalline nature of synthesized nanostructures. The wide band gap nanomaterial was modified by making composites. The fabricated modified nanomaterial has a number of applications, exhibited higher crystallinity and outstanding photocatalytic activity due to the increased surface area after composite formation. The increased surface area was revealed by the electrochemical active Surface area analysis. Furthermore, the nanostructures display tremendous and marvelous properties toward charge storage, as demonstrated via cyclic voltammetry analysis. The synthesized material is suitable for blue LEDs employed by the International Commission on Illumination (CIE).
